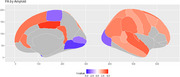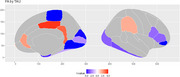# Impact of regional amyloid and tau on diffusion fractional anisotropy at the gray/white interface in sporadic Alzheimer disease

**DOI:** 10.1002/alz70862_110070

**Published:** 2025-12-23

**Authors:** David A. Hoagey, Nicole S. McKay, Qing Wang, Brian A. Gordon, Tammie L.S. Benzinger

**Affiliations:** ^1^ Washington University in St. Louis, St. Louis, MO USA; ^2^ Washington University in St. Louis, School of Medicine, St. Louis, MO USA; ^3^ Washington University in St. Louis School of Medicine, St. Louis, MO USA; ^4^ Washington University School of Medicine, Saint Louis, MO USA

## Abstract

**Background:**

Clinical pathology and staging of Alzheimer disease (AD) is characterized by the temporal progression of amyloid accumulation, tau deposition, and eventually cortical neurodegeneration. Despite this temporal ordering, research is mixed regarding the regional impact of amyloid and tau on neurodegeneration. Diffusion magnetic resonance imaging (MRI) has proven to be sensitive to changes in tissue microstructure demonstrating the capability to detect early presymptomatic changes, track conversion across symptomatic stages, and distinguish between AD subtypes. Here we analyze the impact of regional amyloid and tau on diffusion metrics within the gray/white interface to assess microstructural aspects of neuronal health.

**Method:**

Participants were recruited by the Knight Alzheimer Disease Research Center at Washington University School of Medicine. Data from a total of 354 participants have been processed across T1‐weighted, diffusion MRI, and amyloid and tau positron emission tomography (PET). Participants had an average age of 68.13 years, with 57.9% (205) being female, 87.6% (310) identifying as white, and 15.8% (56) having a Clinical Dementia Rating greater than 0. General linear models assessed the regional effects of amyloid and tau Standardized Uptake Value Ratio SUVR with corresponding FA values.

**Result:**

Results show regionally differential effects on FA for each of amyloid and tau burden. Increases in amyloid have a broad impact on diffusion across many of the higher‐order association cortices, with a pattern comparable to that of typical amyloid deposition. Specifically, we observed increased FA in the frontal, parietal, and superior temporal cortices with higher amyloid burden. In contrast, tau accumulation had a much more focused impact. Increased tau was related to decreasing FA in medial temporal, occipital, and lateral frontal areas, similar to typical patterns of tau spread.

**Conclusion:**

Microstructural features of neuronal health, as proxied from diffusion imaging, are highly associated with the spread of protein biomarkers in AD. Regional patterns appear to mimic the spread of amyloid and tau in presymptomatic stages of sporadic AD. Results could provide an early indicator of changes to myelin integrity or cytoarchitectural fiber complexity reflecting initial neurodegenerative processes These findings help to disentangle the neurodegenerative aspects of AD by improving the biological specificity of neurodegeneration as a biomarker.